# Projected changes in area of the Sundarban mangrove forest in Bangladesh due to SLR by 2100

**DOI:** 10.1007/s10584-016-1769-z

**Published:** 2016-08-16

**Authors:** Andres Payo, Anirban Mukhopadhyay, Sugata Hazra, Tuhin Ghosh, Subhajit Ghosh, Sally Brown, Robert J. Nicholls, Lucy Bricheno, Judith Wolf, Susan Kay, Attila N. Lázár, Anisul Haque

**Affiliations:** 1 University of Southampton, Southampton, SO17 1BJ UK; 2 School of Oceanographic Studies, Jadavpur University, Kolkata, 700 032 India; 3 Tyndall Centre for Climate Change Research, Norwich, UK; 4 National Oceanography Centre, Liverpool, L3 5DA UK; 5 Plymouth Marine Laboratory, Plymouth, PL1 3DH UK; 6 Bangladesh University of Engineering and Technology, Dhaka, 1000 Bangladesh

**Keywords:** Digital Elevation Model, Tidal Flat, Mangrove Forest, National Wetland Inventory, Mangrove Area

## Abstract

The Sundarbans mangrove ecosystem, located in India and Bangladesh, is recognized as a global priority for biodiversity conservation and is an important provider of ecosystem services such as numerous goods and protection against storm surges. With global mean sea-level rise projected as up to 0.98 m or greater by 2100 relative to the baseline period (1985–2005), the Sundarbans – mean elevation presently approximately 2 m above mean sea-level – is under threat from inundation and subsequent wetland loss; however the magnitude of loss remains unclear. We used remote and field measurements, geographic information systems and simulation modelling to investigate the potential effects of three sea-level rise scenarios on the Sundarbans within coastal Bangladesh. We illustrate how the Sea Level Affecting Marshes Model (SLAMM) is able to reproduce the observed area losses for the period 2000–2010. Using this calibrated model and assuming that mean sea-level is a better proxy than the SLAMM assumed mean lower low water for Mangrove area delineation, the estimated mangrove area net losses (relative to year 2000) are 81–178 km^2^, 111–376 km^2^ and 583–1393 km^2^ for relative sea-level rise scenarios to 2100 of 0.46 m, 0.75 m and 1.48 m, respectively and net subsidence of ±2.5 mm/year. These area losses are very small (<10 % of present day area) and significantly smaller than previous research has suggested. Our simulations also suggest that erosion rather than inundation may remain the dominant loss driver to 2100 under certain scenarios of sea-level rise and net subsidence. Only under the highest scenarios does inundation due to sea-level rise become the dominant loss process.

## Introduction

The Sundarbans are the world’s largest single block of mangrove forest, and a world heritage site, spanning the India-Bangladesh border. Apart from being a unique ecosystem, mangroves (and other wetlands) provide substantial protection to coastal populations from flooding, erosion and natural hazards (Das and Vincent 2009; Wolanski 2006). In more developed countries e.g., UK, USA and Netherlands, natural vegetation which had previously been destroyed is being reinstated, in some cases as an ecosystem-based flood defence (Stijn et al. 2013). Although mangrove tree species are able to tolerate inundation by tides, they can die and their former habitat can convert to tidal flats or open water when sea-level rise (SLR) causes the frequency and duration of inundation to exceed species-specific physiological thresholds (Ball 1988). With global mean SLR projected up to 0.98 m or greater (albeit with lower probability) in 2100 relative to 1985–2005 (Church et al. 2013), the Sundarbans, with a mean elevation approximately 2 m above mean sea-level (MSL) is under threat from inundation and loss.

The Sundarbans mangrove forest is an ensemble of different mangrove species the spatial distribution of which is likely to change by the end of the century. Mukhopadhyay et al. (2015) suggested that the spatial distribution of Sundarbans mangrove species assemblages will alter substantially within a hundred years if historical trends are projected forward. The areal distribution of some of the dominant freshwater loving species assemblages like Sundari (Heritiera), Goran (Ceriops), Passur (Xyocarpus) would decrease with concomitant increase of more salt tolerant species like Gewa (Excoecaria), Keora (Sonneratia),Kankra (Bruguiera). In this paper we will refer to the ensemble of species as mangroves and are interested on Mangrove area changes.

The relative threat from SLR for the Sundarbans remains unclear. In a global scale assessment, Gilman et al. (2008) suggested that anthropogenic stressors rather than relative SLR (RSLR) can account for most of the average annual rate of mangrove loss world-wide at present, estimated to be 1–2 %/year, but RSLR may constitute a substantial proportion of predicted future losses by 2100, on the order of 10–20 % but, analysis at the Sundarbans scale suggested RSLR being the main driver of future mangrove area losses. For example, Huq et al. (1995), in a preliminary analysis with very limited information, suggested that “*one meter rise in mean sea level will probably lead to the destruction of the Sundarbans by its complete inundation*”. Loucks et al. (2010) estimated that most of the Bangladesh Sundarbans area will be below MSL if a 0.28 m increase above the MSL in the year 2000 occurs in the next 50–90 years, assuming no change in the current local conditions (i.e., the net increase in elevation remains in the range 4–7.8 mm/year). Lovelock et al. (2015) developed a model (hereafter referred to as the “Lovelock model”) to predict the time to submergence of a mangrove ecosystem subject to accelerated SLR based on the concept of the loss of “elevation capital”, the potential of a mangrove ecosystem to remain within a suitable inundation regime (between Highest Astronomical Tide and MSL). The Lovelock model suggests that the Sundarbans, where there is a relatively high tidal range (4 m) and high sediment supply, will persist beyond 2100 even under the most severe SLR scenario (1.4 m by 2100). The elevation capital approach used in the Lovelock model is better aligned with the known ecophysiology of mangrove forest (e.g., ability to cope with salt and to maintain water and carbon balance and tidal dispersal of propagules) than an estimation just based on MSL rise (i.e., no tidal range included). However, it does not include important processes such as tides, subsidence and coastal erosion due to storms that also influence the mangrove area changes. The aim of this work is to explore how plausible changes in SLR might influence the mangrove forest area of the Bangladesh Sundarbans and test the earlier conclusions about their high vulnerability to SLR.

This paper analyses the magnitude of the net area changes of the Sundarbans by 2100 under three different SLR scenarios. In particular, it combines the latest Digital Elevation Model (DEM) available for the study zone (IWM 2009) and the Sea Level Affecting Marshes (SLAMM) numerical model (Park et al. (1989), http://warrenpinnacle.com/prof/SLAMM/) to explore the possible area change in response to different SLR scenarios. Three SLR scenarios have been explored in this study, based on the Fifth Assessment Report of the Intergovernmental Panel on Climate Change (Pachauri et al. 2014) combined with estimates of net subsidence (i.e., combined effect of land sinking and land rising, including sedimentation) from Brown and Nicholls (2015). In the results section, it is shown how the SLAMM model is able to reproduce the observed mangrove area loss for the period 2000–2010. Assuming a representative wave-induced erosion rate, the model is then used to assess future mangrove area changes by 2100. Finally, the results obtained from this work are discussed and compared with other projections and their implications considered.

## Methodology

### Study area

**Fig. 1:**
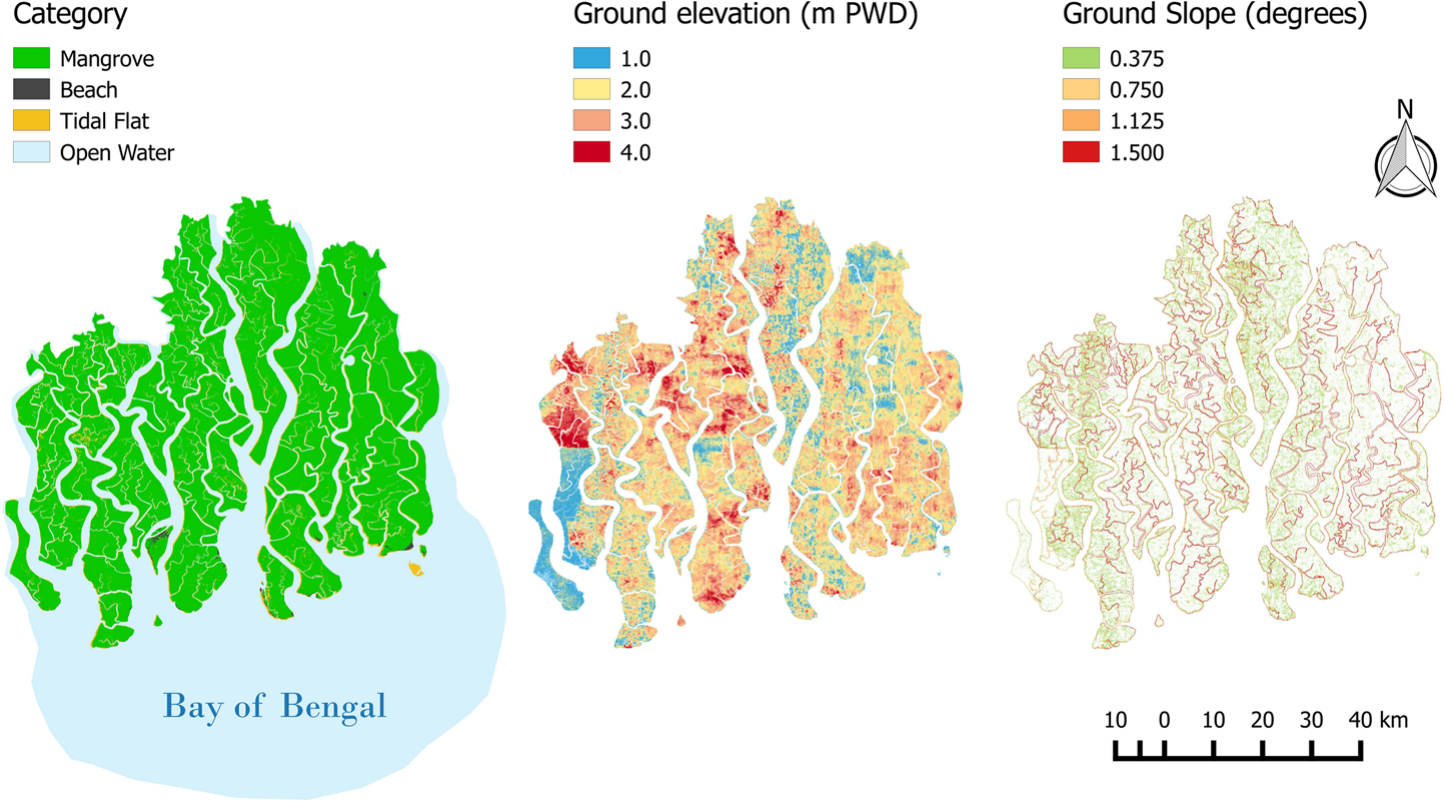
Main wetland categories, ground elevation and slope of the study zone (PWD stands for Public Works Datum)

The study area is located at the northern end of the Bay of Bengal, which has a tropical monsoonal climate with average annual rainfall of 1800 mm and average annual maximum and minimum temperatures 30 °C and 21 °C respectively. The Sundarbans forest consists of 10,200 km^2^ of coastal area of which 5,937 km^2^ and 4,263 km^2^ of protected Reserve forests are located in Bangladesh and India, respectively (Nandy and Kushwaha 2010). This study focuses on the Bangladesh Sundarbans (Fig. 1). The entire Sundarbans is limited at its landward side by embankments that protect polders and their coastal population from flooding, preventing inland migration and potentially squeezing the mangrove area. The mangrove forest is the most abundant geomorphic unit in the Sundarbans, with a mean elevation of about 2 m above MSL, but other geomorphic units such as tidal flats and estuarine beaches are also present.

### Sea level affects marshes model

Change in the area of the Sundarbans in response to SLR is modelled using the “Sea Level Affecting Marshes Model” (SLAMM version 6.2 beta-64bit, http://warrenpinnacle.com). The SLAMM model assumes that wetlands inhabit a range of vertical elevations that is a function of the tidal range. Within SLAMM, there are five primary processes (inundation, erosion, overwash, saturation, accretion) but in this study we have only considered inundation, erosion and accretion. Saturation – upland migration of coastal swamps and fresh marshes as a response of the fresh water table rising- is excluded since coastal swamps and fresh marshes categories are not present on the study zone and the Sundarbans are surrounded by dikes and agricultural land, limiting the mangroves’ uplands migration. Overwash is also excluded as there are no beaches. Estuarine beaches, which are present at the Sundarbans, are assumed to erode at the same user defined erosion rate as tidal flats:

- Inundation: The rise of water levels and the salt boundary are tracked by reducing elevations of each cell as sea levels rise, thus keeping Mean Tide Level (hereinafter referred to as MTL) constant at zero. Spatially variable effects of land subsidence or isostatic rebound are included in these elevation calculations. The effects on each cell are calculated based on the minimum elevation and slope of that cell.
- Erosion: Erosion is triggered based on a threshold of maximum fetch (9 km) and the proximity of the marsh to estuarine water or the open ocean. When these conditions are met, horizontal erosion occurs at a rate based on site-specific data. Horizontal erosion rates may be specified as a function of marsh type and may be specified to vary spatially using “subsite polygons.” For each site or subsite, erosion parameters for tidal flats, marshes, and swamps may be specified. Tidal-flat erosion rates pertain to both tidal flats and estuarine beaches (if the beach has adequate fetch to trigger erosion). The tidal-flat erosion parameter also pertains to ocean beaches if the specific beach erosion module is not switched on.
- Accretion: Sea level rise is offset by sedimentation and vertical accretion using average or site-specific values for each wetland category. Accretion rates may be spatially variable within a given model domain.

While SLAMM includes wetland categories other than mangrove forest, mangroves is the only wetland type considered for tropical coastal systems (tropical coastal systems are defined in the model as sites containing 0.5 % or more total land coverage by mangroves, SLAMM 6.2, Technical documentation, page 40). Therefore, in tropical systems, such as the current study case, any land inundated with saline water is assumed to convert to a mangrove forest. Inundation is induced by changes in the relative SLR (i.e., the balance of subsidence, sedimentation and Eustatic SLR). SLAMM assumes that wetland categories are in quasi-equilibrium with SLR and only one change of category is allowed at each time step. A time step of 10 years has been used to simulate the period 2000 to 2100. Table 1 shows the full list of inputs and parameters used for the simulation of Sundarbans area changes under the three different sea-level scenarios. The most important SLAMM input data are the Digital Elevation Model (DEM) and the National Wetland Inventory (NWI). The NWI contains the dominant wetland category for each cell (SLAMM has a database with definitions of 23 categories).

**Table 1:** SLAMM inputs type, value and source and site parameters used for the SLAMM simulations

Inputs	Value	Source
NWI Photo Date (year)	2003/2005	Mukhopadhyay et al. 2015
DEM Date (year)	2006/2007	IWM 2009
Time step (years)	1	
Cell width (m)	30	IWM 2009
Direction Offshore of DEM	South	
Ground Elevation Trend (mm/year)	+2.5, 0, −2.5	Brown and Nicholls (2015)
Elevation of DEM relative to PWD (m)	0.459	IWM 2009
Great Diurnal Tide Range (m)	0	
SLR (eustatic) above present values by 2100 (m)	0.46, 0.75, 1.48	
Tidal Flat Erosion (horz. m/year)	20	Rahman et al. (2011); Sarwar and Woodroffe (2013)
Use of elev pre-processor [True,False]	FALSE	

The NWI was produced using the year 2003/2005 updated Geological Survey of Bangladesh (GSB) (Mukhopadhyay et al. 2015). Figure 1 shows the main SLAMM-wetland categories identified within the study case: mangroves (3,778 km^2^), tidal flats (238 km^2^) and estuarine beaches (12.5 km^2^). Tidal flats have the lowest mean elevation, followed by estuarine beaches and mangroves. Surrounding water is defined as estuarine open water (category 17 in the SLAMM database). Erosion is triggered based on sufficient fetch (the distance across which wind-driven waves can be formed). If the fetch is greater than 9 km, the model assumes horizontal erosion at a given user defined rate (Knutson et al. 1981). As the fetch for the Sundarbans is much greater than 9 km, so the seaward limit has to be chosen far enough from the seaward margin of the Sundarbans to guarantee that the threshold fetch is reached along the entire sea coast.

The DEM used in this study has been provided by the Institute for Water Modelling of Bangladesh (IWM 2009). Figure 1 shows the high resolution (<1 m accuracy elevation and resolution of 30 m^2^ square cells) ground elevation and slope. The slope data has been calculated from the DEM using QGIS-terrain toolbox. The calculated cell mean slope is 0.28° with the largest slopes of about 2 to 5° at the transition from tidal flat to Mangrove as shown in Fig. 1. The elevation data set used to build the DEM was initially collected by FINNMAP, a Finnish consulting firm, in 1991 for the Government of Bangladesh. The original FINNMAP data are referenced to the “Survey Of Bangladesh” datum (SOB) which is a constant datum approximating present mean sea level. The DEM was updated and provided by IWM (2009). IWM (2009) used Google images from 2006–2007 to correct outdated FINNMAP elevation data and to delineate the Sundarbans forest, providing the final DEM used in this study. It is defined at 30 m resolution and is referred to the PWD datum, the “Public Works Datum” which has been established by the Bangladeshi government at 0.459 m below MSL of the ocean at present day. Geomorphological mapping of the area was based on the Geological Survey of Bangladesh (http://www.gsb.gov.bd/rvedr/images/files/BD_Coast_Geomorphologicalmap.pdf). SLAMM uses the MTL as the internal datum, and therefore elevation data was corrected so that mean tide level is set to zero. In addition to the DEM, the slope of each cell is also provided as an input, since the fraction of the cell that is converted to another wetland class is computed as a function of the fraction of the cell inundated.

The NWI is based on an interpretation of satellite data ranging from 2003–2005 and the DEM is obtained from a correction of the FINMAP 1990’s survey using the interpreted shoreline location from aerial imagery of years 2006–07. When dates of NWI and DEM do not match there may be cell types on the NWI inconsistent with the elevation range resulting from combining the DEM and the tidal range. To get a sense of how many cells’ definitions might be inconsistent with cell elevation, we set the elevation pre-processor on SLAMM to false (turned off) and the model was subsequently run from the NWI-date to the initial baseline year (year 2000). The pre-processor automatically adjusts the cell types to the elevation limits given by the DEM, so the changes observed for the baseline year when the pre-processor is turned off represent cells whose definition was not consistent with elevation. The original NWI input data contains 3,778 km^2^ of mangrove area, but after the model was run to the baseline year this was reduced to 3,767 km^2^. The reduction of about 12 km^2^ is due to some mangrove cells being below the MLLW and therefore converted to water cells. As we show below, this 0.3 % change is relatively small compared with the inter-decadal mangrove area changes. The rationale behind the selection of the GDTR and SLR by 2100, net surface elevation change and tidal flat erosion rate are explained in detail below.

### Sundarbans great diurnal tidal range and mangrove elevation threshold used in SLAMM

The Sundarbans Estuarine System is a macro-tidal delta (mean tidal range >4 m) with predominantly semi-diurnal tides that tend to amplify northwards within the major estuaries and tidal creeks (Pethick and Orford 2013). Observations of the spring tides on the Indian side of the Sundarbans Estuarine System, by Chatterjee et al. (2013), suggest that the degree and rate of amplification are not uniform and follow a complex pattern along the different distributaries, such that the observed spring tidal range for all coastal stations is always larger than 4.5 m and can be as large as 6.7 m. Results from Kay et al. (2015), using a regional ocean model, suggest that the tidal range has little variation along the Sundarbans coastline and over three different epochs in the future up to 2100.

SLAMM was developed primarily for the micro-tidal ranges and predominantly diurnal tides in the US and implicitly assumes that mangroves persist from Mean Lower Low Water (mean of the lower low water height each day, hereinafter referred to as MLLW) as the lower elevation boundary for this category up to an elevation equivalent to the Mean High Higher Water (mean of the higher high water height each day, hereinafter referred to as MHHW). The Great Diurnal Tide Range (difference between MHHW and MLLW) is therefore a key item of input data to assess any mangrove area changes. If a GDTR of 4.5 m is assumed, the lower elevation at which mangroves will be assumed to persist is 2.25 m below MSL. While this value might be adequate for many wetlands species in non-tropical regions, especially under micro tidal conditions, it is unrealistic for the mangrove forest of the Sundarbans. An analysis of the cumulative frequency of elevation (not shown) suggest that the actual MSL (0.549 m above PWD) seems to be a better representation of the lower limit of mangroves in the Sundarbans (i.e., 99 % of Mangroves are above MSL). In this context, and for the SLAMM simulations presented in this paper, it is assumed that GDTR is equal to 0 m for the entire study site (i.e., no amplification along tributaries) and it remains unchanged over time and under different SLR scenarios.

### Relative SLR scenarios

Brown and Nicholls (2015) reviewed net subsidence (i.e., difference between sedimentation, isostatic rebound and subsidence) rates in the Ganges–Brahmaputra–Meghna delta, reporting 205 measurements based on a range of methods and timescales. Subsidence can result from natural processes of geology and soil compaction, as well as anthropogenic effects due to extraction of water and is also affected by the available sediment supply carried by the river system. Compared with other land uses in the delta (e.g., urban or croplands), the Sundarbans reported the lowest mean (2.8 mm/year) and median (2.0 mm/year) rates of net subsidence. Overall, they found the median rate of subsidence preferable to the mean, as a few single large measurements of subsidence skewed results. Brown and Nicholls (2015) also state that the more recent and shorter term measurements reported higher rates of subsidence, either due to anthropogenic influences or as a result of partial measurement. Thus to reflect these differences, a representative value of −2.5 mm/year of net subsidence in the Sundarbans is used in the SLAMM simulations and assumed to be valid to 2100. Simulations are also run using 0 mm/year and +2.5 mm/year to assess the sensitivity of the results to the uncertainty on net subsidence rate.

The projected SLR by 2100 for the three different sea-level scenarios are 0.46 m, 0.75 m and 1.48 m, respectively, for the low, medium and high SLR scenarios. The low and medium scenarios correspond with mean and maximum projections under the RCP4.5 scenario for Haldia, West Bengal (Pachauri et al. 2014); these do not include rapid ice sheet dynamics. The high sea-level scenario corresponds to the H++ scenario range which considers high and plausible, but unlikely scenarios (Jevrejeva et al. 2014): the 95 % value for RCP8.5 (0.98 m) plus 0.5 m due to Antarctica Ice Sheet melting (Levermann et al. 2014). A large value of 1.48 m has been considered to explore a high SLR scenario which is consistent with the extreme scenario of Lovelock et al. (2015).

### Results

**Fig. 2:**
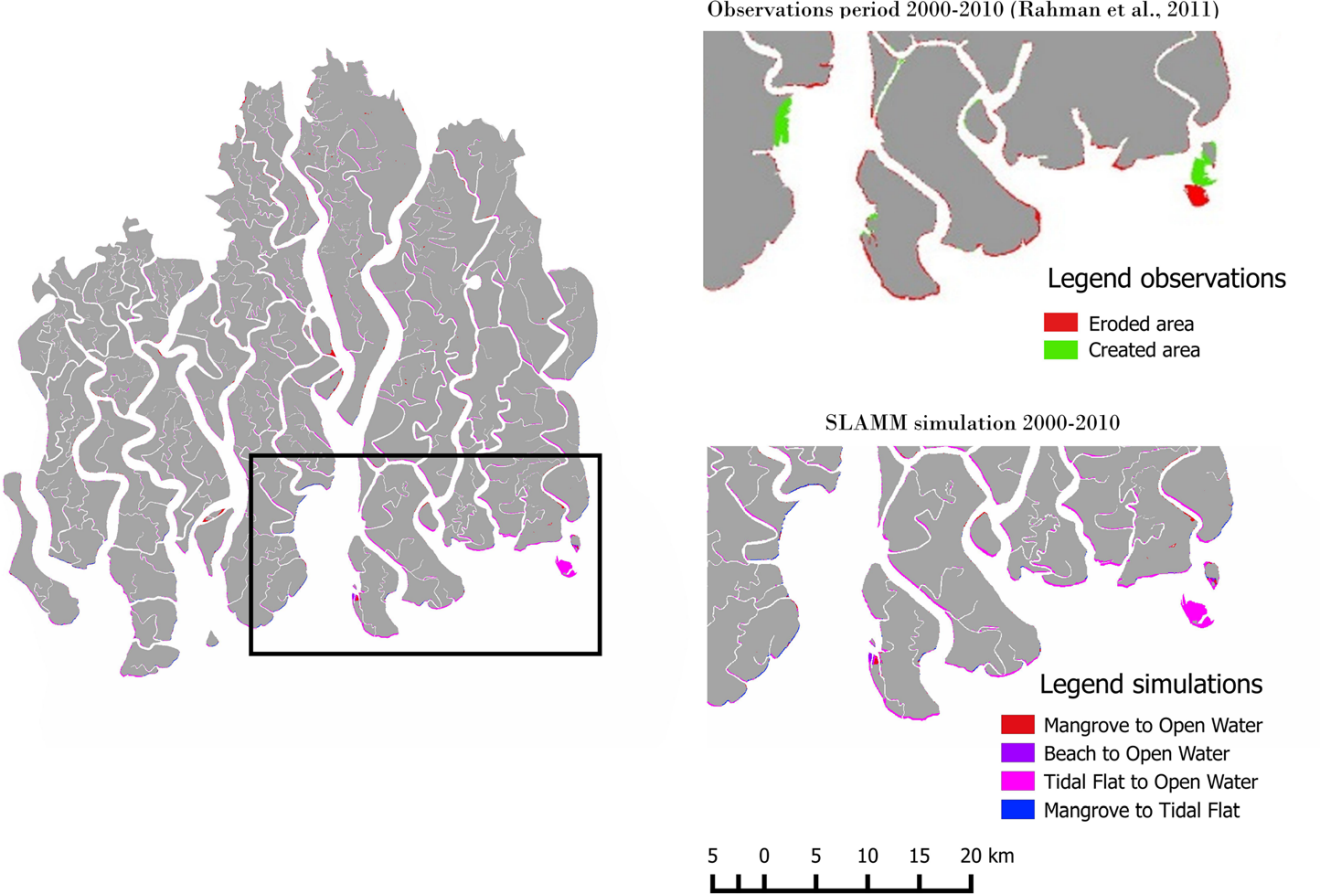
SLAMM is able to reproduce observed wetland area losses by Rahman et al. (2011) for the period 2000–2010. While the main erosion behaviour is captured, there are zones where area has been gained that are not reproduced

The simple decision tree and erosion rule used in the SLAMM model is shown to be capable of reproducing the observed mangrove erosion/conversion for the period 2000 to 2010 (Fig. 2). Rahman et al. (2011) have analysed Landsat images and for the period from 2000 to 2010 showed a net total erosion of 93.5 ± 0.4 km^2^ for the entire Sundarbans (i.e., including the Indian Sundarbans): about half (48.56 km^2^) corresponds with the Bangladeshi Sundarbans. Sarwar and Woodroffe (2013) reported that erosion rates of up to 20 m/year are typical along the Sundarbans coast. The SLAMM model, with a one decade time step and 20 m/year of tidal flat erosion rate, assuming no net subsidence, produces a net erosion of 57 km^2^ of mostly tidal flat (52 km^2^), mangrove (5 km^2^) and estuarine beaches (0.5 km^2^) converted into water cells, which is in good agreement with observations. A closer look at the observations and simulation reveals that while the overall erosion behaviour is qualitatively captured, there are zones where mangrove creation has been observed that the model is not able to reproduce due to the assumption of the uniform net subsidence for the entire study area. The simulation produces realistic erosion along south facing shorelines, but also in sections of the tidal channels where the fetch is large enough. However, observations do not show erosion to the same extent in the major tidal channels.

**Fig. 3:**
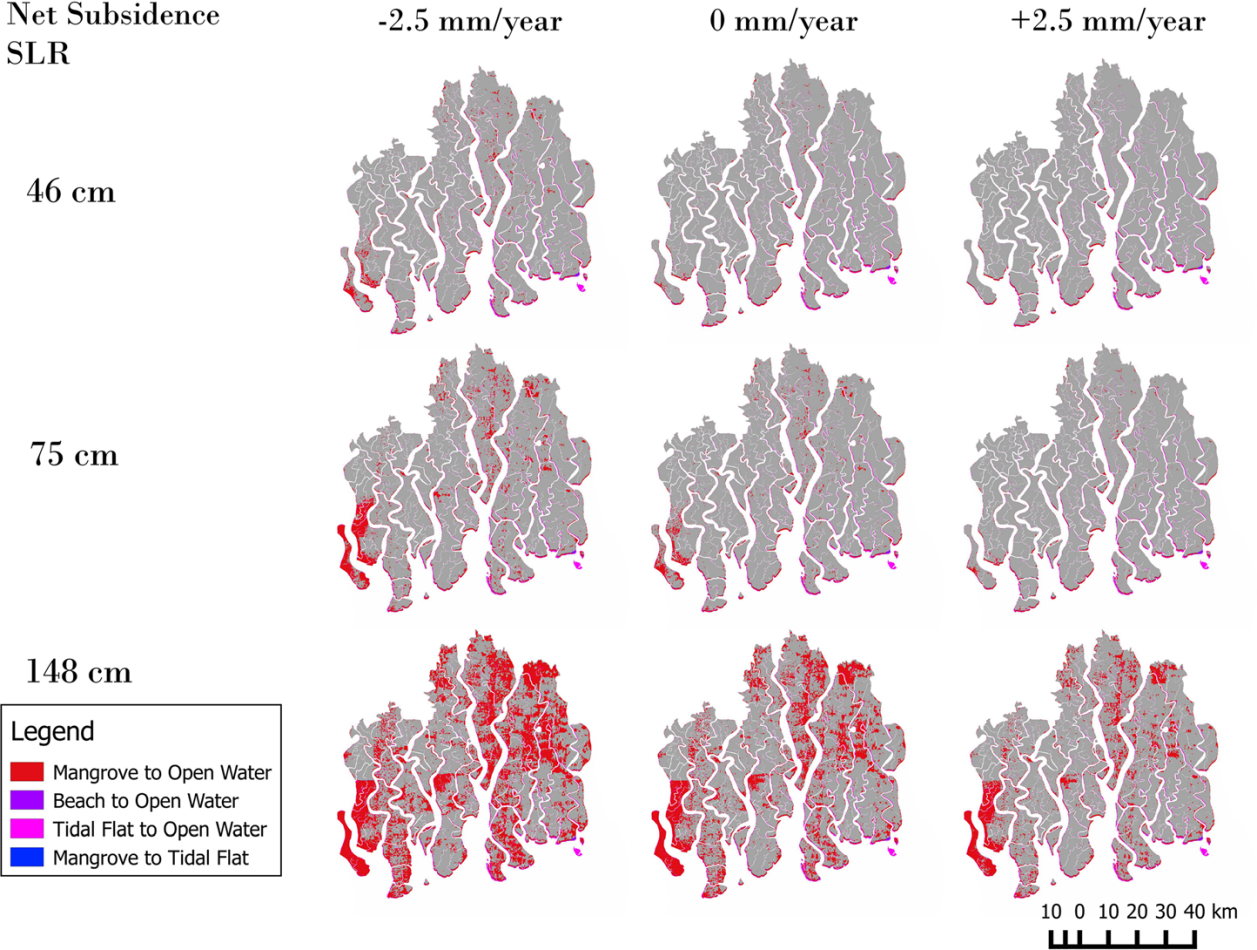
Simulated mangrove area losses by 2100 under nine different RSLR scenarios

Simulated mangrove area changes are minimal for the moderate and medium RSLR scenarios by the end of the 21^st^ century, but significant for the extreme RSLR scenario (Fig. 3). Even assuming the net uplift, the mangrove area is reduced by less than 46 km^2^ by 2050, relative to the baseline year, in the moderate and medium SLR scenarios and by 81 km^2^, 111 km^2^ and 583 km^2^ (9.8 %) by the end of the 21^st^ century for the low, medium and high RSLR scenarios respectively. The Mangrove area reduction increases for the scenarios under no net subsidence and net subsidence of −2.5 mm/year.

The percentage of Mangrove losses in 2100 due to erosion or inundation cannot be distinguished from Fig. 3. To separate erosion from inundation, a simple analysis can be done that combines cell conversion information with ground elevation relative to MTL. Inundated cells are those converted cells for which elevation relative to MSL is below 0 and eroded cells are converted cells for which elevation is still above this threshold. Results of this analysis are shown on Table 2 together with the area changes for all scenarios. Erosion is dominant only in 3 out of the 9 scenarios. Total losses (e.g., erosion and inundation) rates varies more than two order of magnitude from the lowest value of 0.8 km^2^/year for the 45 cm SLR and net uplift up to the largest value of 14 km^2^/year for the 148 cm SLR and net subsidence.

**Table 2:** Simulated mangrove area change and relative importance of inundation and erosion for three different SLR scenarios and net subsidence rates

	Net subsidence rate		
MSL by 2100 m (eustatic)	−2.5 mm/year	0 mm/year	+2.5 mm/year
0.46	−178 km^2^ 65 % In 35 % Er	−103 km^2^ 16 % In 84 % Er	−81 km^2^ 16 % In 84 % Er
0.75	−376 km^2^ 85 % In 15 % Er	−200 km^2^ 69 % In 31 % Er	−111 km^2^ 42 % In 58 % Er
1.48	−1393 km^2^ 97 % In 3 % Er	−927 km^2^ 95 % In 5 % Er	−584 km^2^ 92 % In 8 % Er

*In* Inundation, *Er* Erosion

The sensitivity of the ratio of erosion versus inundation to the assumption of a 20 m/year constant annual erosion rate was found to be minimal. Using an annual erosion rate as high as 100 m/year for the 45 cm SLR scenario only increased the percentage of mangrove cells lost by 2100 due to erosion by 1 % and 2 % for the net uplift and net subsidence scenarios respectively.

**Fig. 4:**
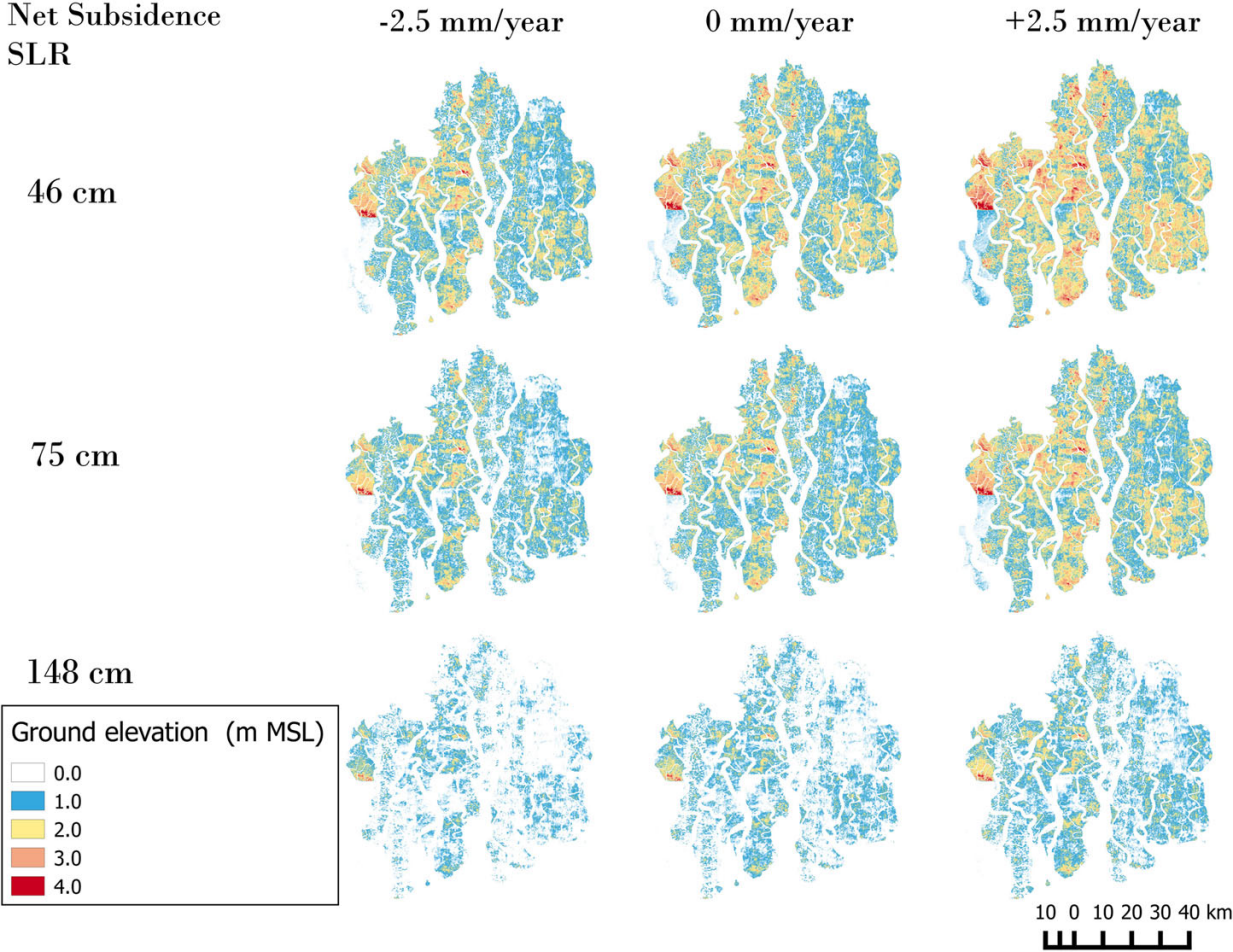
Simulated ground elevation relative to MTL by 2100 under nine different RSLR scenarios

Figure 4 shows the ground elevation relative to the MSL by 2100 for all nine scenarios. Ground elevation is the combination of net subsidence/uplift and eustatic SLR. The simulation results indicate the gradual sinking of the Sundarbans compared to the MSL. Even under the lowest RSLR scenario, the majority of the Sundarbans will become just slightly elevated above the MSL (i.e., <1 m). This sinking trend is intensified under the most extreme RSLR scenario, where the significant land areas are lost by 2100, no matter which subsidence rate is considered.

### Discussion

The simulated mangrove losses due to RSLR by the end of 21^st^ century at the Bangladesh Sundarbans are much smaller than earlier estimates (e.g., Huq et al. 1995; Loucks et al. 2010), which are widely reported in discussions about Bangladesh and SLR. For example, Colette (2007) suggested that a 45 cm rise in global sea level by 2100 might lead to the destruction of 75 % of the Sundarbans mangroves suggesting significant differences on the DEM used in different studies; the DEM used by Loucks et al. (2010) suggest that most of the Sundarbans will be below MSL for a SLR of +28 cm. However, in our study this level of inundation is only observed for the most extreme scenario (Fig. 3). An analysis of the cumulative elevation frequency suggests that even a 2.19 m RSLR above PWD would not bring about the complete inundation of the Sundarbans but would affect about 50 % of the area. This suggest that the DEM used in this study is clearly different to the one used in previous studies. An accurate and up to date DEM of the study zone is still missing and it is key to assess the fate of the Sundarbans by the end of 21^st^ century.

The average rate of annual mangrove loss from simulated mangrove changes for the moderate, medium and high sea-level scenarios and assuming net subsidence of −2.5 mm/year are 1.78 km^2^/year, 3.76 km^2^/year and 14 km^2^/year, respectively, and lower than the suggested 0.2 % per year average annual loss based on results for mangrove areas for the Pacific Islands by Gilman et al. (2008). Our results also differ concerning the mechanism of loss due to SLR. While Gilman et al. (2008) suggested that SLR could be the main driver by the end of 2100, our simulations suggest that for the Bangladesh Sundarbans erosion may remain the dominant driving force by the end of the 21^st^ century depending on the net subsidence rate. Under the net uplift scenario, only in the worst-case scenario does inundation due to SLR become of similar or greater importance to coastal erosion (i.e., the amount of mangrove area loss at the coastal edge and inundation are similar) with the average annual losses (due to both erosion and inundation). Erosion will still dominant by 2100 under no net subsidence and 46 cm SLR. Inundation will be dominant in the rest of the remaining scenarios.

This study has a number of limitations that are important to highlight. There are several processes and process interactions that might influence mangrove area changes but which are not considered in this model, such as the positive feedback between sedimentation and mangrove growth (e.g., Gilman et al. 2008). The assumption of uniform net subsidence (i.e., balance of subsidence and sedimentation) for the entire study area results in SLAMM’s failure to reproduce the localized mangrove accretion relative to the observed period 2000–2010 (Sarwar and Woodroffe 2013). The GDTR is also assumed uniform and constant for the entire study area and simulated period. While the projections of Kay et al. (2015) support the assumption of relative small changes in GTDR over time, the observed amplification along the tributaries is not captured by SLAMM simulations. Any effect of changing coastline on the tidal range is also neglected. Changes in sediment supply, or erosion rate due to cyclones are also not considered. While this study focuses on the influence of RSLR, the authors acknowledge that there are anthropogenic causes that are inducing mangrove area losses today and may induce mangrove area losses in the future. For example, oil spills from ships that travel through the Sundarbans via the Passur River to Mongla port have caused mangrove losses in this century (Rahman et al. 2009).

### Conclusion

The SLAMM model has been applied for the first time to the Sundarbans of coastal Bangladesh to assess the impact of three SLR scenarios. MSL is assumed as a better proxy for lower elevation at which Mangroves can be developed than MLLW and 0 m GDTR has been used for the entire study area. Using the best DEM and NWI available to date, the impact of SLR, including processes such as net subsidence and erosion due to wave action, on mangrove area losses has been estimated to be lower than previous researchers have suggested. Our simulations suggest that the key driving force of mangrove losses in the Bangladesh Sundarbans will very much depend on the net subsidence rate. Under a net uplift scenario of 2.5 mm/year is erosion, not inundation the main driver under SLR of 46 cm and 75 cm by 2100. However, for the SLR scenario of approximately 1.5 m/century, inundation due to SLR becomes dominant. The study gives a new and important perspective on the impact of SLR on the world’s largest single block of mangrove forest and a world heritage site, which appears to be more resilient to SLR than earlier analyses suggested but which trend will very much depend on the uncertain net subsidence rate. Building on this study, further more detailed investigations of the Sundarbans would be useful.
